# Fenofibrate Improves Renal Lipotoxicity through Activation of AMPK-PGC-1α in *db/db* Mice

**DOI:** 10.1371/journal.pone.0096147

**Published:** 2014-05-06

**Authors:** Yu Ah Hong, Ji Hee Lim, Min Young Kim, Tae Woo Kim, Yaeni Kim, Keun Suk Yang, Hoon Suk Park, Sun Ryoung Choi, Sungjin Chung, Hyung Wook Kim, Hye Won Kim, Bum Soon Choi, Yoon Sik Chang, Cheol Whee Park

**Affiliations:** 1 Division of Nephrology, Department of Internal Medicine, Korea University Guro Hospital, Seoul, Korea; 2 Division of Nephrology, Department of Internal Medicine, The Catholic University of Korea, Seoul, Korea; 3 Department of Rehabilitation Medicine, The Catholic University of Korea, Seoul, Korea; Pennington Biomedical Research Center, United States of America

## Abstract

Peroxisome proliferator-activated receptor (PPAR)-α, a lipid-sensing transcriptional factor, serves an important role in lipotoxicity. We evaluated whether fenofibrate has a renoprotective effect by ameliorating lipotoxicity in the kidney. Eight-week-old male C57BLKS/J *db/m* control and *db/db* mice, divided into four groups, received fenofibrate for 12 weeks. In *db/db* mice, fenofibrate ameliorated albuminuria, mesangial area expansion and inflammatory cell infiltration. Fenofibrate inhibited accumulation of intra-renal free fatty acids and triglycerides related to increases in PPARα expression, phosphorylation of AMP-activated protein kinase (AMPK), and activation of Peroxisome proliferator-activated receptor γ co-activator 1α (PGC-1α)-estrogen-related receptor (ERR)-1α-phosphorylated acetyl-CoA carboxylase (pACC), and suppression of sterol regulatory element-binding protein (SREBP)-1 and carbohydrate regulatory element-binding protein (ChREBP)-1, key downstream effectors of lipid metabolism. Fenofibrate decreased the activity of phosphatidylinositol-3 kinase (PI3K)-Akt phosphorylation and FoxO3a phosphorylation in kidneys, increasing the B cell leukaemia/lymphoma 2 (BCL-2)/BCL-2-associated X protein (BAX) ratio and superoxide dismutase (SOD) 1 levels. Consequently, fenofibrate recovered from renal apoptosis and oxidative stress, as reflected by 24 hr urinary 8-isoprostane. In cultured mesangial cells, fenofibrate prevented high glucose-induced apoptosis and oxidative stress through phosphorylation of AMPK, activation of PGC-1α-ERR-1α, and suppression of SREBP-1 and ChREBP-1. Our results suggest that fenofibrate improves lipotoxicity via activation of AMPK-PGC-1α-ERR-1α-FoxO3a signaling, showing its potential as a therapeutic modality for diabetic nephropathy.

## Introduction

Diabetic nephropathy an important and serious complication of diabetes is the most common and most rapidly growing cause of end-stage renal disease creating an enormous expense associated with renal replacement therapy [1]. Glomerular and tubular toxicity resulting from hyperglycemia (glucotoxicity) have been evaluated extensively at the molecular level as contributing factors [2], [3]. Recently, several investigators have shown accumulation of lipids in the kidneys of diabetic humans and experimental models, and have proposed that lipotoxicity and oxidative stress may play an important role in the pathogenesis of diabetic kidney disease, although the underlying molecular mechanisms remain elusive [4]–[6].

Peroxisome proliferator-activated receptor-α (PPARα) is a member of the nuclear hormone receptor superfamily of ligand-activated transcription factors and plays an important role in lipid metabolism [7] as well as sustaining the balance between energy production and utilization in tissues with a high oxidative capacity, such as the liver, kidney and heart [8]. PPARα activation attenuates or inhibits several mediators of vascular injury, including lipotoxicity, inflammation, reactive oxygen species (ROS) generation, endothelial dysfunction, angiogenesis and thrombosis in type 2 diabetes and high fat diet-induced renal damage [9]–[11], which are all associated with AMP-activated protein kinase (AMPK) and endothelial nitric oxide synthase (eNOS) [12], [13]. Moreover, PPARα activates peroxisome proliferator-activated receptor γ co-activator (PGC)-1α/β and its key downstream effector, estrogen-related receptor (ERR)-1α, which induces the expression of energy metabolism genes and enhances the mitochondrial oxidative capacity to provide defense against oxidative stress [14], [15]. In addition, the increased activity of sterol regulatory element-binding protein-1 (SREBP-1) and carbohydrate regulatory element binding protein-1 (ChREBP-1) and decreased activity of phosphorylated acetyl-CoA carboxylase (pACC) most likely also play a role in increased free fatty acid (FFA) synthesis and accumulation of triglycerides (TG) in diabetic kidney disease and PPARα activation can suppress the SREBP pathway through the reduction of liver X receptor (LXR)/retinoid X receptor (RXR) formation in the liver [16].

AMPK is a cellular energy sensor, regulated by the ATP/AMP ratio. In muscle and the liver, AMPK increases PGC-1α expression and also directly phosphorylates PGC-1α, increasing its transcriptional activity [17], [18]. Among several transcription factors associated with PPARα, PGC-1α acts as a ‘molecular switch’ in pathways controlling glucose homeostasis [19], and may be a critical link in the pathogenesis of type 2 diabetes. PGC-1α functionally interacts with transcriptional factors, particularly with members of nuclear receptor families such as PPARα, PPARγ, ERR-1α, LXR and hepatocyte nuclear factor-4 α (HNF-4α), although also with non-nuclear receptor transcription factors and regulatory elements including the cAMP response element-binding protein (CREB) and SREBP-1c [20]. These diverse members of the nuclear receptor superfamily, such as PPARα, PPARγ, and ERR-1α, improve hepatic lipogenesis via suppression of the lipogenic transcription factor SREBP-1c [21]. PGC-1α targets ERR-1α [22], which serves as an internal ‘amplifier’ of the PGC-1α cascade and is an important regulator of mitochondrial energy transduction pathways, including fatty acid oxidation and oxidative phosphorylation. In addition, PGC-1α regulates class O forkhead box (FoxO) 3a, which is a direct transcription regulator of a group of oxidative protection genes in primary endothelial cells. FoxO3a and PGC-1α interact directly and cooperatively; their interaction regulates mitochondrial oxidative stress [23].

We have previously reported that PPARα deficiency appears to aggravate the severity of diabetic nephropathy through increase in extracelluar matrix formation, inflammation and circulating FFA and TG concentrations [9]. We also demonstrated that fenofibrate ameliorated diabetic nephropathy directly, which may go beyond a systemic lipid-lowering effect, as evidenced by improvements in albuminuria, glomerular hypertrophy and mesangial expansion in a type 2 diabetic model [10]. However, the underlying mechanisms responsible for the beneficial effect of fenofibrate on diabetic nephropathy are not completely understood. We hypothesized that fenofibrate can potentially improve renal lipotoxicity-induced oxidative stress and apoptosis by way of the activation of AMPK-PGC-1α-ERR-1α and its downstream PI3K-Akt-FoxO3a pathway.

## Materials and Methods

### Experimental methods

Eight-week-old male C57BLKS/J mice were purchased from Jackson Laboratories (Bar Barbor, ME, USA). Male C57BLKS/J *db/m* and *db/db* mice were divided into four groups and received either a regular diet of chow or a diet containing fenofibrate. Fenofibrate (0.1%, w/w, Sigma, St Louis, MO, USA) was mixed into the standard chow diet and provided to *db/db* mice (n = 8) and age and gender-matched *db/m* mice (n = 8) for 12 weeks starting at 8 weeks of age [10]. Control *db/db* mice (n = 6) and control *db/m* mice (n = 6) received normal mouse chow. In total, 125–150 mg/kg/day of fenofibrate was administered to the treated *db/db* and *db/m* groups. At week 20, all animals were anesthetized by intraperitoneal injection of a mixture of Rompun 10 mg/kg (Bayer Korea, Ansan, Gyeonggi-Do, Korea) and Zoletil 30 mg/kg (Virbac, Carros, France). For measurement of the 24-hr urinary protein, the mice were placed in individual mice metabolic cages (Nalgene, Rochester, NY, USA).

The mice were sacrificed and their kidneys removed. All experiments, including Western blots were performed with both renal cortex and medullar. The kidneys were rapidly dissected and stored in buffered formalin (10%) for subsequent immunohistochemical analyses. Blood was collected from the left ventricle and the plasma was stored at −70°C for subsequent analyses. The Animal Care Committee of the Catholic University of Korea approved the experimental protocol and the experiments were performed in accordance with our institutional animal care guidelines.

### Ethics statement

The Animal Care Committee of the Catholic University of Korea approved the experimental protocol (Approval number: Catholic University St.Vincent Hospital 11–016), and the experiments were performed in accordance with our institutional animal care guidelines.

### Measurement of serum parameters

All blood samples were obtained after overnight fasting. The fasting blood glucose was measured by an Accu-check meter (Roche Diagnostics, St Louis, Mo, USA). The HbA1c was determined from red cell lysates by HPLC (Bio-Rad, Richmond, CA, USA). Total cholesterol, TG and FFA concentrations were measured by an auto-analyzer (Wako, Osaka, Japan).

### Assessment of renal function and oxidative stress and the intra-renal lipids

A 24-hr urine collection was obtained using metabolic cages at 20 weeks and urinary albumin concentrations were measured by an immunoassay (Bayer, Elkhart, IN, USA). Plasma and urine creatinine concentrations were measured using HPLC (Beckman Instruments, Fullerton, CA, USA). To evaluate the oxidative stress, we measured 24-h urinary 8-epi-prostaglandin F_2á_ (8-epi-PG F_2á_; OxisResearch, Foster City, CA, USA). The kidney lipids were extracted by the method of Bligh and Dyer, with slight modifications, as previously described (Waco, Osaka, Japan).

### Light microscopic study

Kidney samples were fixed in 10% buffered formalin and embedded in paraffin. The histology was assessed following hematoxylin-eosin staining and periodic acid-Schiff staining (PAS). The mesangial matrix and glomerular tuft areas were quantified for each glomerular cross-section using PAS-stained sections as previously reported [11]. More than 30 glomeruli which were cut through the vascular pole, were counted per kidney and the average was used for analysis.

### Immunohistochemistry for transforming growth factor-β1 (TGF-β1), type IV collagen (Col IV), F4/80, and TUNEL assay

We performed immunohistochemistry for TGF-β1, Col IV and F4/80 as well as a terminal deoxynucleotidyl transferase dUTP nick end labeling (TUNEL) assay. Briefly described, small blocks of kidney were immediately fixed in 10% buffered formalin for 24 h before being embedded in paraffin. Four-micrometer-thick sections of renal tissues were incubated overnight with anti-TGF-β1 (1∶100; R&D Systems, Minneapolis, MN, USA), anti-COL IV (1∶200; Biodesign International, Saco, ME, USA), and anti-F4/80 (1∶200; Serotek, Oxford, UK) in a humidified chamber at 4°C. The antibodies were localized with a peroxidase-conjugated secondary antibody and by using the Vector Impress kit (Vector Laboratories, Burlingame, CA, USA) and a 3,3-diamninobenzidine substrate solution with nickel chloride enhancement. The sections were then dehydrated in ethanol, cleared in xylene and mounted without counterstaining. All of these sections were examined in a blinded manner using light microscopy (Olympus BX-50, Olympus Optical, Tokyo, Japan). For the quantification of the proportional areas of staining, approximately 20 views (400x magnification) were used, randomly located in the renal cortex and the corticomedullary junction of each slide (Scion Image Beta 4.0.2, Frederik, MD, USA). Detection of apoptotic cells in the formalin-fixed, paraffin-embedded tissue was performed by in situ TUNEL, using the ApopTag In Situ Apoptosis Detection Kit (Chemicon-Millipore, Billerica, MA, USA). The TUNEL reaction was assessed in the whole glomeruli biopsy under 400x magnification.

### Western blot analysis and measurement of phosphatidylinositol 3-kinase (PI3K) and phosphorylated acetyl-CoA carboxylase (pACC) enzyme activity in kidney tissue

The total proteins of the renal cortical tissues were extracted with a Pro-Prep Protein Extraction Solution (Intron Biotechnology, Gyeonggi-Do, Korea), following the manufacturer's instructions. Western blot analysis was performed to further confirm the responses using antibodies that recognize the specific epitope. Western blots were carried out for PPARα, total AMPK, phosphorylated (phospho)-Thr^172^ AMPK, PGC-1α, ERR-1α, SREBP-1, ChREBP-1, PI3K, total Akt, phospho-Ser^473^ Akt, total FoxO3a, phospho-Ser^253^ FoxO3a, total FoxO1, phospho-Ser^256^ FoxO1, B cell leukaemia/lymphoma 2 (BCL-2), BCL-2-associated X protein (BAX), Cu/Zn superoxide dismutase (SOD1) and Mn superoxide dismutase (SOD2). Kidney tissues were homogenized in the presence of protease inhibitors to obtain extracts of kidney proteins for the measurement of PI3K activity. Protein concentrations were determined using Bradford reagent (Bio-Rad., Hercules, CA, USA). The amount of phosphatidylinositol-3,4,5-triphosphate (PIP3) produced was quantified by PIP3 competition enzyme immunoassays according to the manufacturer's protocol (Echelon, Inc., Salt Lake City, UT, USA). We also measured the activity of pACC using a mouse pACC ELISA kit (QAYEE-BIO, Shanghai, China).

### In vitro study

For evaluation of the intracellular pathways and apoptosis, mesangial cells were grown in 5 mmol/l D-glucose (low glucose), 30 mmol/l D-glucose (high glucose), or 5 mmol/L D-glucose plus 25 mmol/l mannitol (as an osmotic control for 30 mmol/l D-glucose). NMS2 mesangial cells were used between passages 25 and 30 and transferred to six-well culture plates at a density of 5×10^4^ cells per well. After reaching 60% confluence these cells were maintained in a quiescent state in RPMI with 0.1% fetal bovine serum for 48 hrs. The mesangial cells were then exposed to low glucose, high glucose, or the mannitol control, with or without the additional 24-hr application of fenofibrate (10 µM). Small interfering (siRNA), targeted to AMPKα1, AMPKα2, and PGC-1α, and scrambled siRNA (siRNA cont) were complexed with transfection reagent (G-Fectin; Genolution, Seoul, Korea), according to the manufacturer's instructions. The sequences of the siRNAs were as follows: α1-AMPK, GCAUAUGCUGCAGGUAGAU; α2-AMPK, CGUCAUUGAUGAUGAGGCU; PGC-1α, AAGACGGATTGCCCTCATTTG; and nonspecific scrambled siRNA, CCUACGCCACCAAUUUCGU (Bioneer, Daejeon, Korea). NMS cells in six-well plates were transfected with a final concentration of 100 nM α1- and α2-AMPK siRNAs for 48 hrs by transfection reagent (G-Fectin), according to the manufacturer's instructions. Forty-eight hours after transfection, cells were treated with fenofibrate. The cultured mesangial cells were also transfected with 50 nM control siRNA (cont), or 50 nM of AMPKα1, AMPKα2, or PGC-1α siRNA, and subsequently stimulated by fenofibrate in high-glucose media to evaluate the effects of siRNAs on mesangial cell reactions.

### Statistical analysis

The data are expressed as the mean ± standard deviation (SD). Differences between the groups were examined for statistical significance using ANOVA with Bonferroni correction using SPSS version 19.0 (SPSS, Chicago, IL, USA). A *P* value of <0.05 was considered statistically significant.

## Results

### Physical and biochemical characteristics in *db/m*, *db/m* Feno, *db/db*, and *db/db* Feno mice

The body weight was heavier for the diabetic mice groups than that of the non-diabetic mice groups. Kidney weight, blood glucose, HbA1c, serum total cholesterol, TG and FFA were significantly higher for the *db/db* mice groups than the *db/m* mice groups. There were no differences in the serum creatinine levels among all study groups. There was significantly increased albuminuria in the *db/db* mice compared with those in the *db/m* and *db/m* Feno mice. Fenofibrate treatment ameliorated albuminuria and decreased the urine volume to the levels of the *db/m* and *db/m* Feno mice. Increased creatinine clearance in the *db/db* mice compared with those in the *db/m* and *db/m* Feno mice were decreased to the levels of the *db/m* and *db/m* Feno mice (Table 1).

**Table 1:** Biochemical and physical characteristics of the four groups at the end of the 12 weeks experimental period.

Characteristics	*db/m* Control	*db/m* Feno	*db/db* Control	*db/db* Feno
Body weight (g)	31.6±1.6	28.7±2.0	56.3±4.3***	59.8±2.3***
Kidney weight (g)	0.20±0.02	0.21±0.02	0.27±0.05*	0.27±0.04*
FBS (mg/dl)	168.3±2.7	168.8±17.0	550.5±64.9***	371.2±48.0***
Hb A1c (%)	4.2±0.3	4.4±0.1	8.2±1.1***	6.2±0.5***
Total cholesterol (mg/dl)	106±19	110±14	129±14*	124±23*
Triglycerides (mg/dl)	107±32	101±23	171±27**	167±21**
Free fatty acid (mEg/l)	0.69±0.18	0.71±0.15	1.22±0.21**	1.24±.11**
24 hr albuminuria (ug/day)	12.8±8.0	26.6±7.1	289.8±104.3***	75.4±21.2
Urine volume (ml)	1.0±0.4	1.3±0.4	21.0±9.7***	2.1±0.6
Serum Cr (mg/dl)	0.11±0.02	0.13±0.76	0.09±0.04	0.1±0.04
Cr clearance (ml/min)	0.30±0.17	0.28±0.11	0.65±0.25**	0.40±0.25
Food intake (g/d)	3.2±0.4	3.2±0.8	7.5±1.3***	6.8±1.0***

Cr, Creatinine; FBS, fasting blood sugar, Data are means ± SD. n = 6–8 in each groups.

*P<0.05,

**P<0.01,

***P<0.001 compared with other groups.

### Effects of fenofibrate on the renal phenotypes, TGF-β1, Col IV, and F4/80

**Figure 1:**
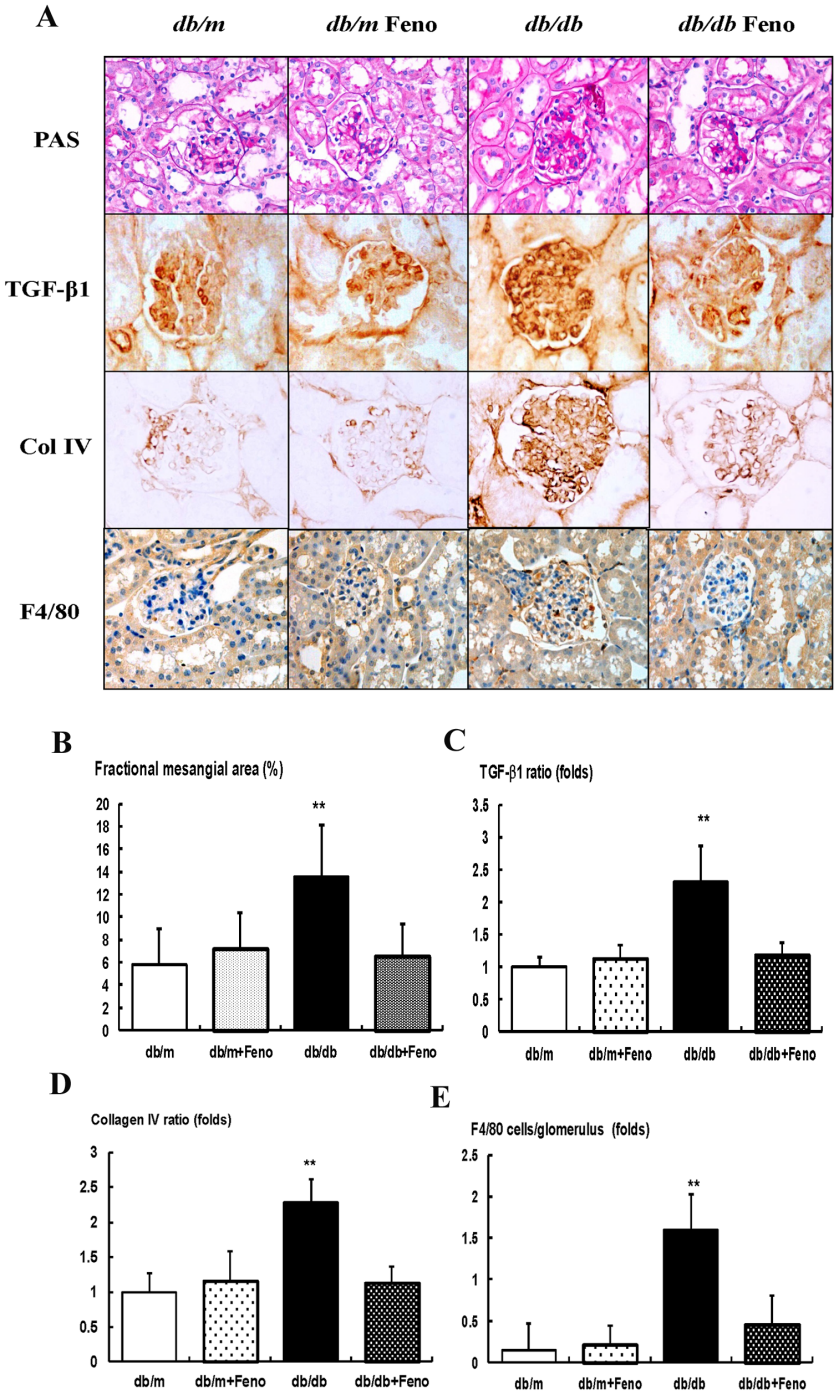
Changes in glomerular phenotypes in fenofibrate-treated *db/db* mice. Glomerular mesangial fractional area, TGF-β1 and collagen IV (Col IV) expression, and a F4/80-positive cell infiltration in the glomerulus in the cortical area of the *db/m* and *db/db* mice, with or without fenofibrate treatment. A) Representative sections stained with periodic acid-Schiff reagent and representative immunohistochemical staining for TGF-β1, type IV collagen, and F4/80-positive cells (dark brown) are shown (original magnification 400x). B-E) Quantitative analyses of the results for the mesangial fractional area (%), TGF-β1, type IV collagen, and F4/80-positive cells (fold) are shown. Abbreviations: Col IV, type IV collagen; PAS, periodic acid-Schiff stain, **p<0.01 *vs.* the *db/m* Cont (*db/m*), *db/m* Feno (*db/m+Feno*), and *db/db* Feno (*db/db+Feno*) mice.

There were no significant differences of fractional mesangial area between the *db/m* and *db/m* Feno mice (Fig. 1*A*). In contrast, there was a significant increase in the mesangial area in the *db/db* mice as compared to the *db/m* mice (*P*<0.01). Consistent with the changes of the mesangial fractional area, the expression of the pro-fibrotic growth factor TGF-β1, which is associated with extracellular matrix Col IV and inflammatory cell infiltration in the glomerular area were significantly increased in the *db/db* mice as compared to the *db/m* and *db/m* Feno mice (Fig. 1*B–E*). All of the diabetes-induced renal phenotypic changes and inflammation seen in the *db/db* mice were ameliorated by the fenofibrate treatment.

### Renal cortical expressions of PPARα, phospho-Thr^172^ AMPK and total-AMPK, PGC-1α, ERR-1α, SREBP-1, ChREBP-1, pACC, and intra-renal TG and FFA

**Figure 2:**
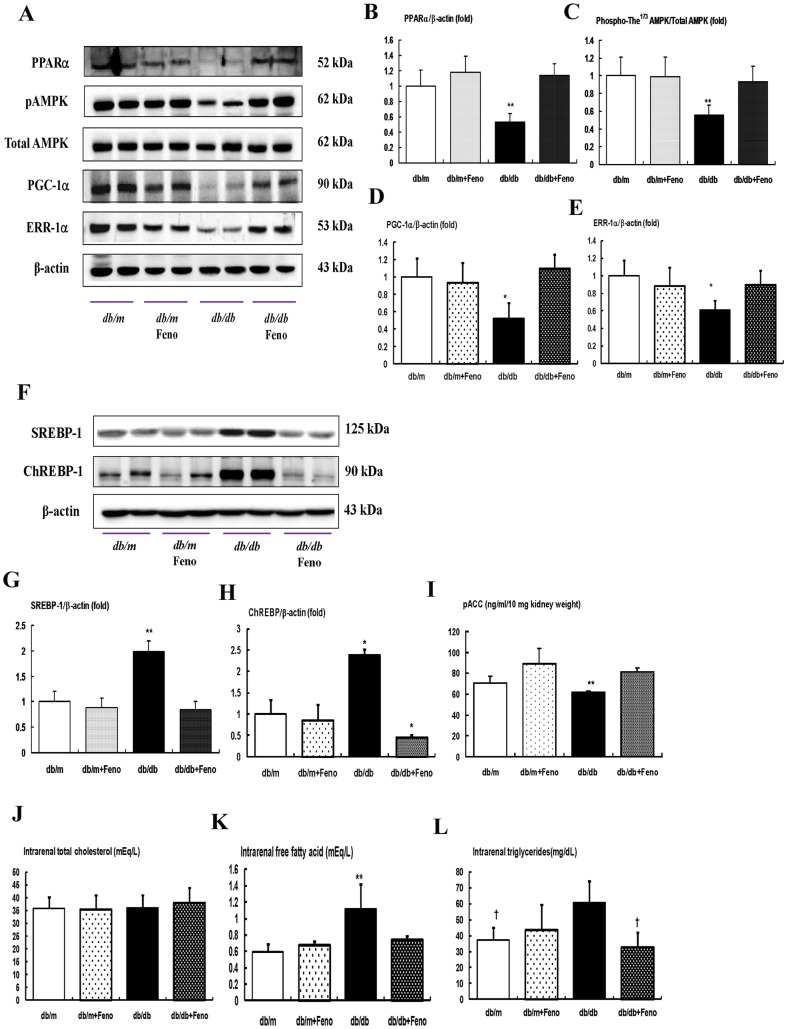
PPARα, Phospho-AMPK Thr^172^, total AMPK, PGC-1α, ERR-1α, SREBP-1, ChREBP-1, and intra-renal lipid levels in the renal cortex of the *db/m* and *db/db* mice with or without fenofibrate treatment. Protein lysates (30 µg) from renal cortex were separated by SDS-PAGE and analyzed by Western blotting. A) Representative Western blotting is shown for PPARα, phospho-Thr^172^ AMPK, total AMPK, PGC-1α, ERR-1α, and β-actin. B–E) Quantitative analyses are shown for PPARα/β-actin (B), phospho-Thr^172^ AMPK/total AMPK (C), PGC-1α/β-actin (D), and ERR-1α/β-actin (E). *p<0.05 and **p<0.01 *vs.* the *db/m* control, *db/m* Feno, and *db/db* Feno mice. F–H) Representative Western blot of SREBP-1, ChREBP-1 and β-actin, (F) and the corresponding quantitative analyses (G and H). The level of pACC in the kidney are shown (I). J–L) Quantitative analyses of intra-renal total cholesterol (J), free fatty acid (FFA) (K), and triglyceride (L) concentrations. *p<0.05 and **p<0.01 *vs.* the *db/m* control, *db/m* Feno, and *db/db* Feno mice; †† *p*<0.01 *vs*. the *db/m* control and *db/m* Feno mice. Abbreviations: pAMPK, phospho-Thr^172^ AMPK.

As shown in Fig. 2, diabetes markedly decreased the PPARα protein levels in the *db/db* mice compared with that of the *db/m* and *db/m* Feno mice as assessed by Western blot analysis (Fig. 2*B, P*<0.01). Fenofibrate treatment in the *db/db* mice recovered the PPARα levels to the levels of the *db/m* and *db/m* Feno mice. To evaluate the changes of the PPARα target proteins, we examined the phospho-Thr^172^/total-AMPK expression ratio, as well as PGC-1α and ERR-1α expression, in the kidney. The phospho-Thr^172^/total-AMPK ratio was decreased along with PGC-1α and ERR-1α expression which were recovered in the *db/db* mice with fenofibrate treatment (Fig. 2*C–E, P*<0.05, respectively). The levels of SREBP-1 and ChREBP-1 in the kidneys were significantly higher in the *db/db* mice and decreased with fenofibrate treatment (Fig. 2*F–H*). In contrast, the level of pACC in the kidney was lower in the *db/db* mice and fenofibrate treatment significantly increased (Fig. 2*I*). Consistent with the decrease of AMPK-PGC-1α-ERR-1α-pACC and increases of SREBP-1 and ChREBP-1 in the *db/db* mice, direct measurement of the intra-renal lipid concentrations showed that FFA and TG were increased in the *db/db* mice, but not the total cholesterol concentration. These findings were of great interest in that fenofibrate treatment completely restored the suppressed expression of PPARα associated with decreases of the FFA and TG concentrations in the kidneys which were significantly increased owing to diabetes (Fig. 2*J–L, P*<0.05, respectively).

### Renal expression of the PI3K-phospho-Akt-phospho-FoxO3a signaling pathway

**Figure 3:**
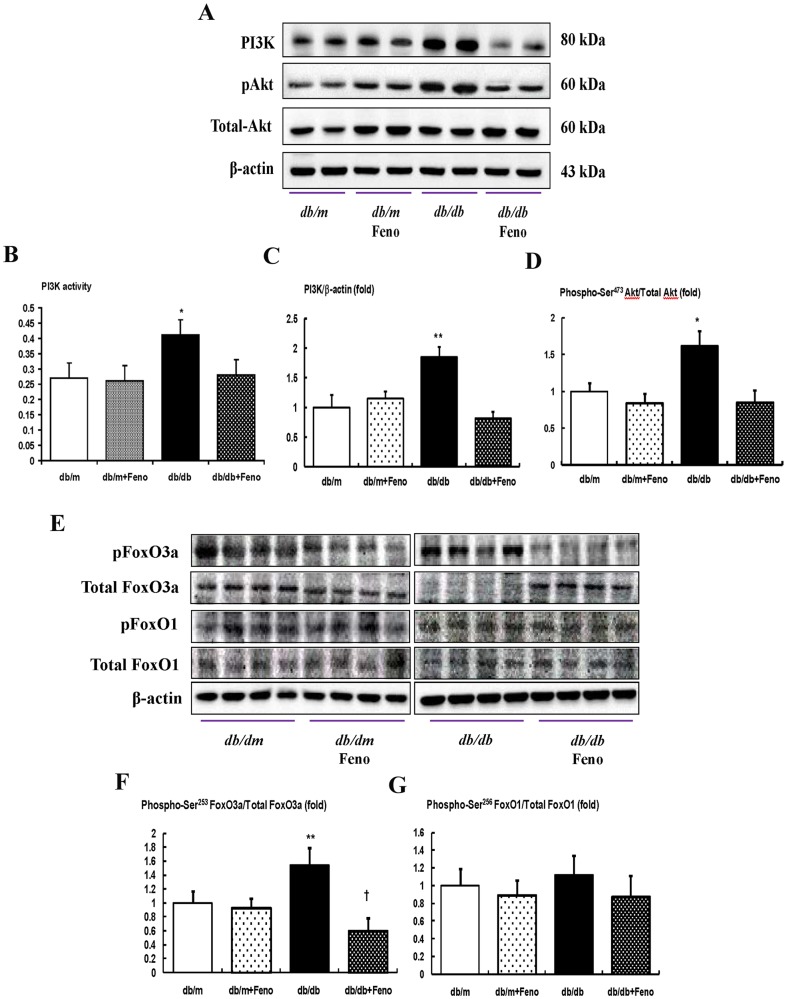
PI3K activity, PI3K, phospho–Akt Ser^472^, total Akt, phospho-FoxO3a Ser^253^ and total FoxO3a, and phospho-FoxO1 Ser^256^ total FoxO1 in the renal cortex of the *db/m* and *db/db* mice with or without fenofibrate treatment. Protein lysates (40 µg) from renal cortex were separated by SDS-PAGE and analyzed by Western blotting. A) Representative Western blot showing PI3K, phospho-Ser^472^ Akt, total Akt, and β-actin levels. B) PI3K activity. C–D) Quantitative analyses of the results from the Western blot: PI3K/β-actin (C); phospho-Ser^472^ Akt/total Akt (D). E) Representative Western blot showing phospho-Ser^253^ FoxO3a, total FoxO3a, phospho-Ser^256^ FoxO1, and total FoxO1 and β-actin levels. F–G) Quantitative analyses of the results from the Western blot: phospho-Ser^253^ FoxO3a/total FoxO3a (F); phospho-Ser^256^ FoxO1/total FoxO1 (G). *p<0.05 and **p<0.01 vs. *db/m* control, *db/m* Feno, and *db/db* Feno mice. † p<0.05 vs. *db/m* control, *db/m* Feno and *db/db* mice. Abbreviations: pAkt, phospho-Ser^472^ Akt; pFoxO1, phospho-Ser^256^; pFoxO3a, phospho-Ser^253^ FoxO3a.

We determined the levels of intra-renal PI3K and phosphate-Ser^473^ Akt, using a Western blot analysis and assessing PI3K activity by ELISA. The PI3K level and activity, as well as the phospho-Ser^473^/total-Akt ratio, were significantly increased in the *db/db* mice compared with the *db/m* and *db/m* Feno mice (Fig. 3*A–D, P*<0.01 and <0.05, respectively), whereas the fenofibrate treatment in the *db/db* mice normalized the PI3K level and phospho-Ser^473^/total-Akt ratio to those levels of the *db/m* or *db/m* Feno mice. An increased expression of phospho-Ser^253^ FoxO3a was noted in the *db/db* mice compared with the *db/m* and *db/m* Feno mice (*P*<0.01). Treatment with fenofibrate markedly decreased the expression of phospho-FoxO3a Ser^253^ in the *db/db* mice, which resulted in an increase in the total FoxO3a (Fig. 3*F, P*<0.05). On the contrary, the protein expression of phospho-Ser^256^ FoxO1 was mildly increased in *db/db* mice, but there was no significant difference between those with or without the fenofibrate treatment (Fig. 3*G*).

### Renal expression of pro-apoptotic BAX, anti-apoptotic BCL-2, expression and TUNEL-positive cells

**Figure 4:**
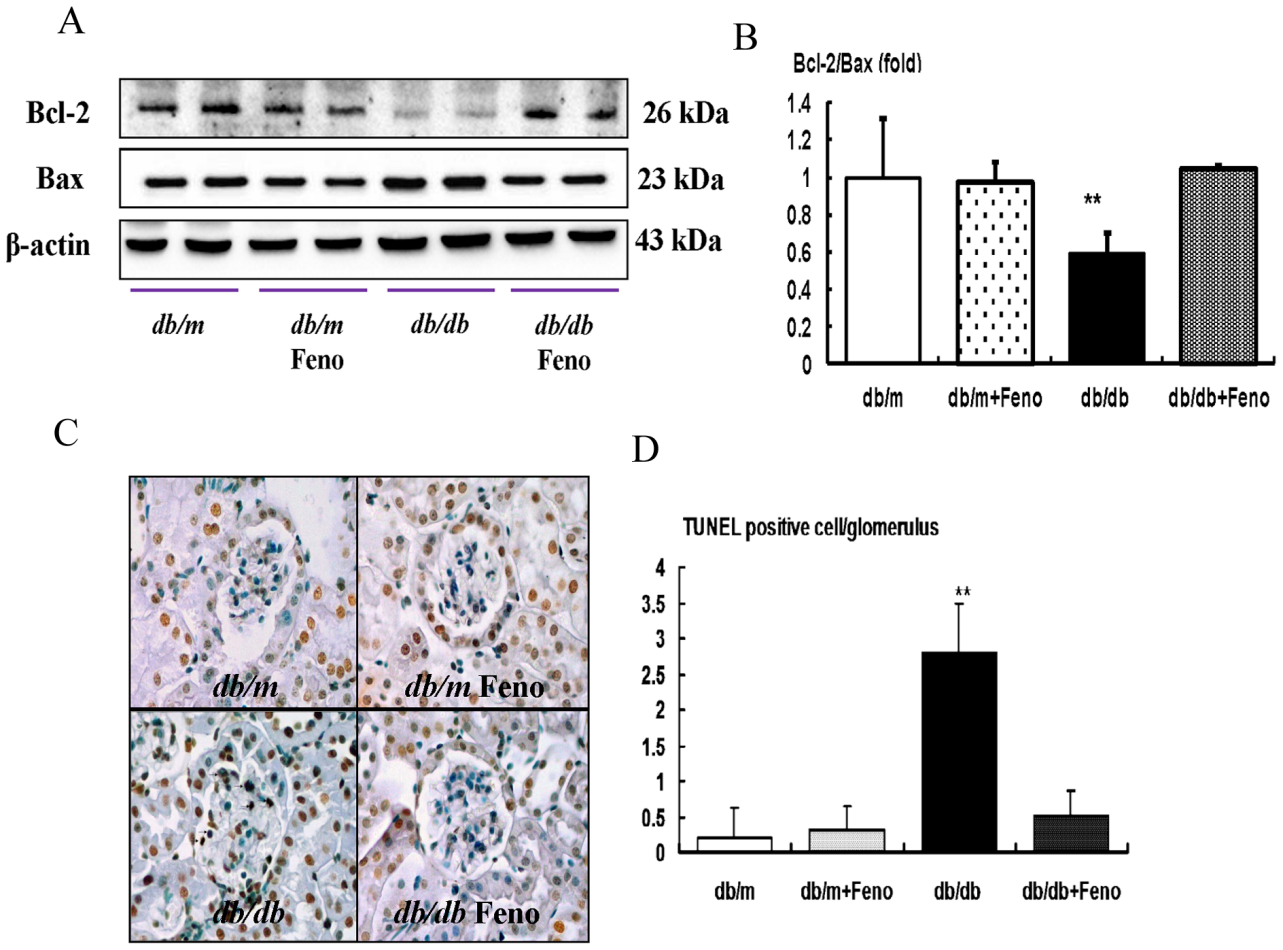
Pro-apoptotic BAX, anti-apoptotic BCL-2, and TUNEL assay in the renal cortex of *db/m* and *db/db* mice with or without fenofibrate treatment. Protein lysates (40 µg) from renal cortex were separated by SDS-PAGE and analyzed by Western blotting. A) Representative Western blot analysis of the BAX, BCL-2, and β-actin levels. B) The quantitative analyses of the results of the BCL-2/BAX ratio are shown. C) Representative immunohistochemical staining for TUNEL-positive cells and the quantitative analyses of the results are shown. **p<0.01 vs. *db/m* control, *db/m* Feno, and *db/db* Feno mice.

It is well known that FoxO3a activation has anti-stress and anti-apoptotic activities by enhancing the BCL-2 activity and down-regulating the pro-apoptotic BAX activity. Consistent with the changes of FoxO3a, the BAX protein level was increased in the *db/db* mice, whereas the BCL-2 protein level was decreased in the *db/db* mice compared with those of the *db/m* and *db/m* Feno mice. Consequently, the BCL-2/BAX ratio was significantly decreased in the *db/db* mice. Fenofibrate treatment in the *db/db* mice increased the BCL-2 protein, and it decreased the Bax protein, which resulted in a normalized level of the ratio of the BCL-2/BAX expression (Fig. 4*B, P*<0.01). To investigate lipotoxicity-induced apoptosis, we studied the renal TUNEL-positive cells from all experimental groups. Compared to the *db/m* and *db/m* Feno mice, there was a significant increase in the number of TUNEL-positive cells in the glomerulus of the *db/db* mice, and the number of TUNEL-positive cells was decreased by fenofibrate treatment (Fig. 4*C–D, P*<0.01).

### Effects of fenofibrate on the intra-renal SOD1, SOD2, and 24-hr urinary 8-isoprostane concentrations

**Figure 5:**
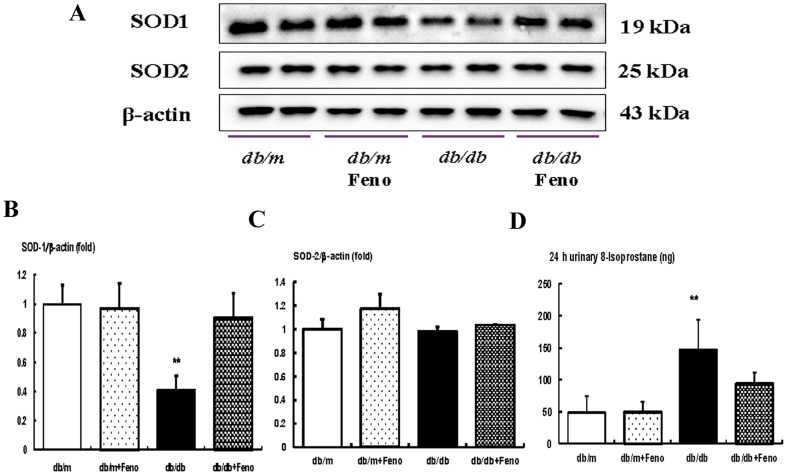
Intra-renal SOD1, SOD2, and 24-hr urinary 8-isoprostane concentrations in *db/m* and *db/db* mice with or without fenofibrate treatment. Protein lysates (10 µg) from renal cortex were separated by SDS-PAGE and analyzed by Western blotting. A–C) Representative Western blot analysis of the SOD1 and SOD2 and the quantitative analyses of the results are shown: SOD1 (B) and SOD2 (C). D) The 24-hr urinary 8-isoprostane concentrations of the study mice are shown. *p<0.05 and **p<0.01 vs. the *db/m* control, *db/m* Feno, and *db/db* Feno mice. Abbreviations: Cont, control.

We investigated the changes to the SOD1 and SOD2 content associated with FoxO3a activation. SOD1 levels were significantly lower in the *db/db* mice than in the *db/m* and *db/m* Feno mice. Fenofibrate treatment in the *db/db* mice returned the SOD1 to the levels of those from *db/m* and *db/m* Feno mice (Fig. 5*A–C*). Increases in the renal oxidative stress and lipid peroxidation were observed in the *db/db* mice compared with those levels of the *db/m* and *db/m* Feno mice with respect to the urinary 8-isoprostane concentration (Fig. 5*D, P*<0.01). On the other hand, the 24-hr urinary 8-isoprostane concentration was significantly decreased in the *db/db* mice undergoing fenofibrate treatment compared with that of the control groups. These findings suggest that oxidative stress in the *db/db* mice could be ameliorated by fenofibrate treatment.

### In vitro studies

**Figure 6:**
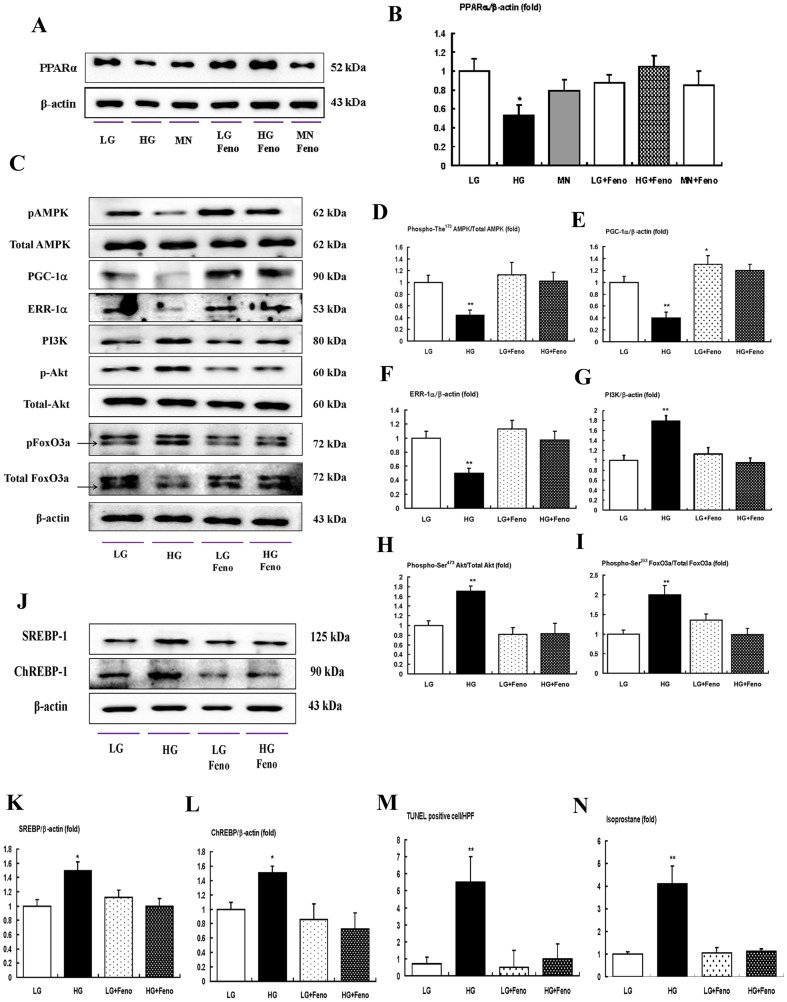
The effect of fenofibrate on intracellular signaling, apoptosis, and oxidative stress (8-isoprostane concentrations in the cultured media) in the mesangial cells cultured in low-glucose (5 mmol/l D-glucose) or high-glucose (30 mmol/l D-glucose) conditions with or without fenofibrate treatment (50 µg/ml). PPARα, Phospho-Thr^172^ AMPK, total AMPK, PGC-1α, ERR-1α, PI3K, phospho-Ser^472^ Akt, total Akt, phospho-Ser^253^ FoxO3a, total FoxO3a, SREBP-1, and ChREBP-1 levels were assessed in the cultured mesangial cells. Protein lysates (10 µg) were separated by SDS-PAGE and analyzed by Western blotting. A) Representative Western blot analysis and quantitative analyses of PPARα are shown. C-I) Representative Western blot analysis of phospho-Thr^172^ AMPK, total AMPK, PGC-1α ERR-1α, PI3K, phospho-Ser^472^ Akt, total Akt, phospho-Ser^253^ FoxO3a, and total FoxO3a and β-actin levels in the cultured mesangial cells and the quantitative analyses of the results are shown; phospho-Thr^172^ AMPK, total AMPK (D); PGC-1α (E); ERR-1α (F); PI3K (G); phospho-Ser^472^ Akt, total Akt (H); phospho-Ser^253^ FoxO3a and total FoxO3a (I). J-L) Representative Western blot analysis of SREBP-1 and ChREBP-1 in cultured mesangial cells and the quantitative analyses of the results. M–N) The quantitative analyses of the TUNEL-positive cells (M) in the cultured mesangial cells and 8-isoprostane concentrations (N) from the cell-culture media of the study are shown. *p<0.05 and **p<0.01 compared with low glucose. Abbreviations: HG, high glucose; LG, low glucose; MN, mannitol; pAMPK, phospho-Thr^172^ AMPK; pAkt, phospho-Ser^472^ Akt; pFoxO3a, phospho-Ser^253^ FoxO3a.

**Figure 7:**
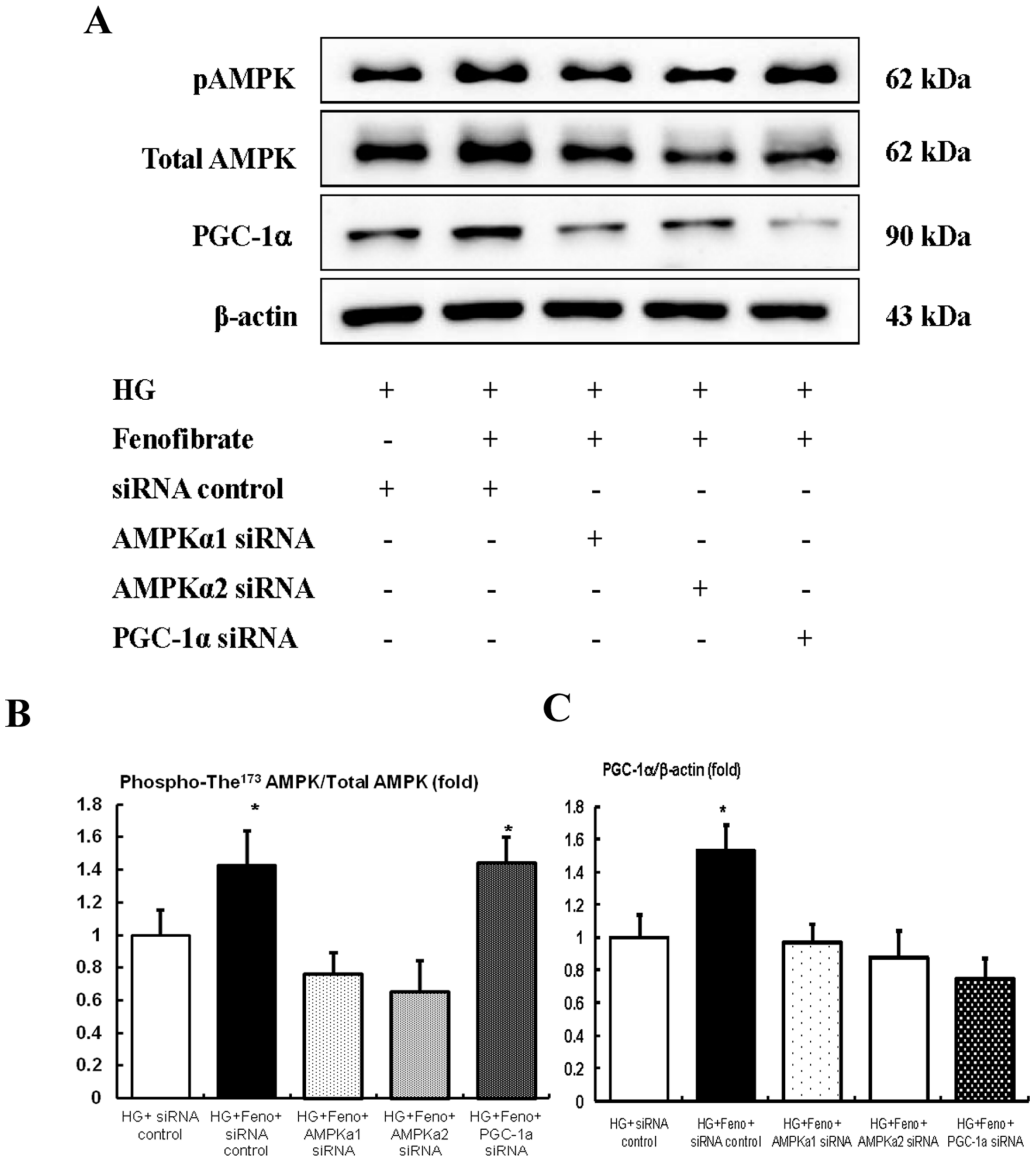
Immunoblot for phospho-AMPK, total AMPK, and PGC-1α in AMPKα1 siRNA, AMPKα2 siRNA, or PGC-1α siRNA knock-down mesangial cells (NMS) in a high-glucose environment with fenofibrate treatment. The cultured mesangial cells were transfected with 50/l control siRNA or 50 nmol/l Ampkα1, Ampkα2, or PGC-1α siRNA using transfection reagent (G-Fectin) and stimulated with fenofibrate in high-glucose media. Approximately 48 hr after transfection, the levels of phospho-Thr^172^ AMPK, total AMPK, SIRT1, and PGC-1α signaling in the high-glucose media stimulated with fenofibrate, were analyzed. Protein lysates (10 µg) from the cultured mesangial cells were separated by SDS-PAGE and analyzed by Western blotting. A–C) Representative Western blot analysis of phospho-Thr^172^ AMPK, total AMPK, PGC-1α, and β-actin levels, (A) and the quantitative analyses of the results are also shown: phospho-Thr^172^ AMPK and total AMPK (B); PGC-1α (C). *p<0.05 compared with other groups. Abbreviations: HG, high glucose; Feno, fenofibrate; pAMPK, phospho-Thr^172^ AMPK.

In diabetic *db/db* mice, mesangial matrix expansion with an increased number of apoptotic mesangial cell deaths is one of the characteristic phenotypes in relation to the oxidative stress. As fenofibrate reversed the diabetes-induced detrimental renal effects in *db/db* mice, we evaluated the effects of fenofibrate on high glucose-induced oxidative stress and the effects of apoptosis related to the AMPK-PGC-1α-ERR-1α signaling and their downstream effecters the PI3K-Akt-FoxOs axis, in cultured mesangial cells. High glucose (30 mmol/l of D-glucose) induced significant decreases in the activation of PPARα expression and mannitol-treated mesangial cells suppressed PPARα expression compared with low glucose. Fenofibrate treatment restored PPARα levels in the high-glucose and mannitol group compared with the low glucose group (Fig. 6*A–B, P*<0.05). Furthermore, Western blot analyses showed that high glucose decreased phospho-Thr^172^ AMPK-PGC-1α-ERR-1α and fenofibrate treatment in high-glucose media reversed the activation of phospho-Thr^172^ AMPK-PGC-1α-ERR-1α (Fig. 6*C–F*). Consistent with the change of AMPK-PGC-1α-ERR-1α, high glucose increased the lipogenic enzymes SREBP-1 and ChREBP-1 (Fig. 6*J–L*). High glucose additionally increased the PI3K-phospho-Ser^472^ Akt and, subsequently, phospho-Ser^253^ FoxO3a (Fig. 6*C, G-I*), while fenofibrate prevented high glucose-induced oxidative stress and apoptosis related to the activation of AMPK-PGC-1α-ERR-1α signaling and the suppression of PI3K-Akt phosphorylation and subsequently, the FoxO3a phosphorylation pathway. High glucose additionally increased the number of TUNEL-positive cells and 8-isoprostane concentrations in the cell culture media (Fig. 6*M, N*). When compared with high glucose, the addition of fenofibrate to low levels of glucose did not affect the intracellular signaling and mesangial cell apoptosis. To evaluate whether AMPK or PGC-1α was associated with stimulation by fenofibrate treatment, we performed an additional study using siRNAs for *AMPK α1, AMPK α2*, and *PGC-1α* in cultured mesangial cells. The transfected siRNAs for *AMPK α1* and *AMPK α2* suppressed fenofibrate-induced AMPK-PGC-1α signaling compared with the siRNA control group. However, transfection with *PGC-1α* only suppressed PGC-1α, not the phospho-Thr^172^/total AMPK ratio (Fig. 7*A–C*).

### Expressions of PPARα, phospho-Thr^172^ AMPK and total-AMPK, PGC-1α, and phospho-FoxO3a signaling pathway in the renal medulla

**Figure 8:**
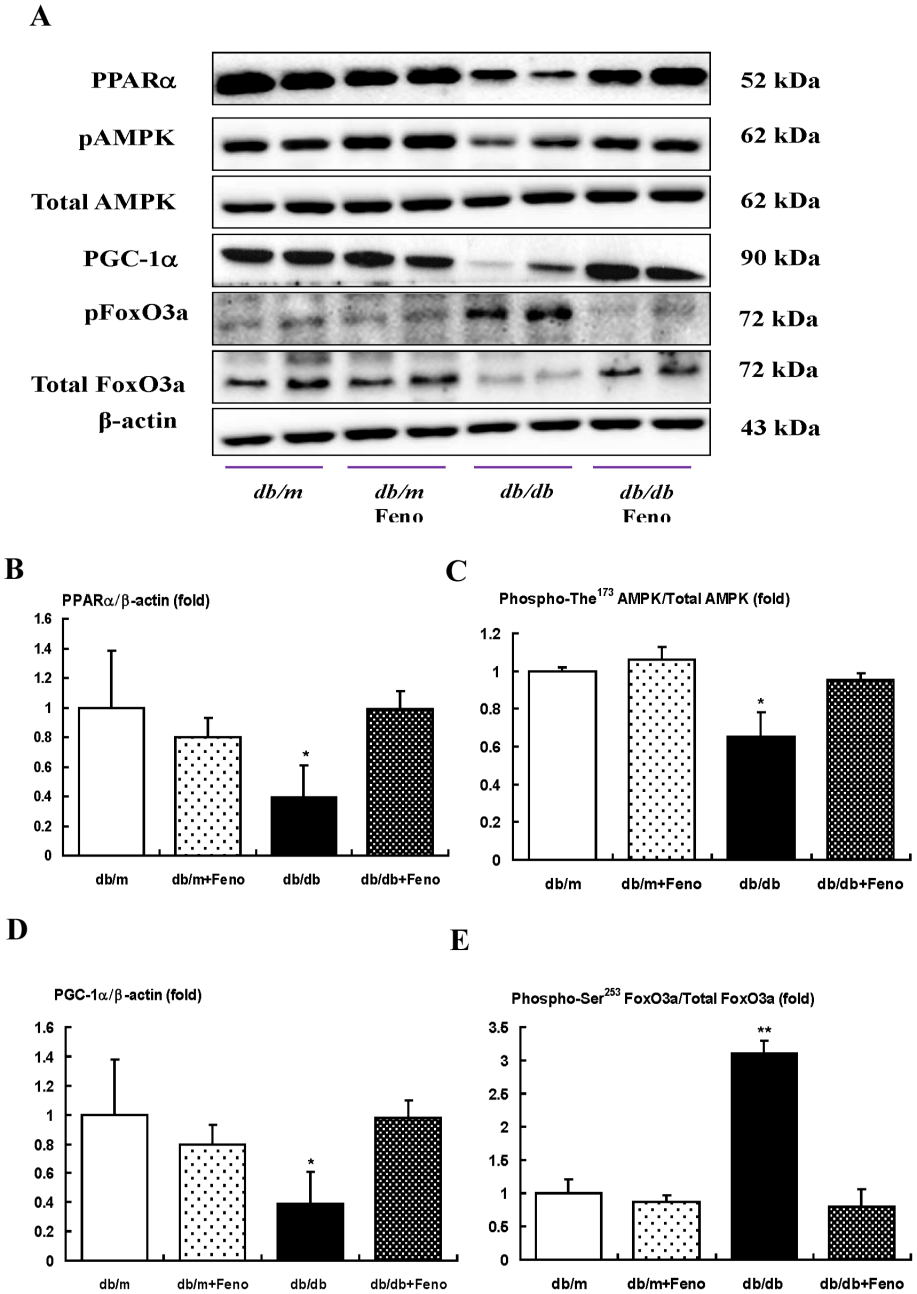
PPARα, Phospho-AMPK Thr^172^, total AMPK, PGC-1α, and phospho-FoxO3a Ser^253^ and total FoxO3a levels in the renal medullar of the *db/m* and *db/db* mice with or without fenofibrate treatment. Protein lysates (30 µg) from renal medullar were separated by SDS-PAGE and analyzed by Western blotting. A) Representative Western blotting is shown for PPARα, phospho-Thr^172^ AMPK, total AMPK, PGC-1α, phospho-FoxO3a Ser^253^, total FoxO3a, and β-actin. B-E) Quantitative analyses are shown for PPARα/β-actin (B), phospho-Thr^172^ AMPK/total AMPK (C), PGC-1α/β-actin (D), and phospho-FoxO3a Ser^253^ and total FoxO3a (E). *p<0.05 and **p<0.01 *vs.* the *db/m* control, *db/m* Feno, and *db/db* Feno mice.

The target molecules of fenofibrate are reported to be different between glomerulus and whole kidney tissues. We investigated the expressions of PPARα, the phospho-Thr^172^/total-AMPK expression ratio, as well as PGC-1α and phospho-Ser^473^/total-Akt ratio in the renal medullar. Interestingly, there was no significant difference in the changes of PPARα- phospho-Thr^172^ AMPK-PGC-1α-phospho-Ser^253^ FoxO3a in the renal medulla compared to those in the renal cortex (Fig. 8).

## Discussion

This study demonstrates that diabetic nephropathy is associated with an increase in renal lipid accumulation, apoptotic renal injury and oxidative stress which are related to a decreased level of PPARα expression in diabetic mice. These changes lead to the inactivation of AMPK-PGC-1α-ERR-1α signaling and the deregulation of their target molecules, SREBP-1, ChREBP-1 and PI3K-Akt-FoxO3a, which subsequently result in an increase in oxidative stress in the kidney. On the contrary, fenofibrate ameliorates diabetic nephropathy by way of the activation of AMPK-PGC-1α-ERR-1α signaling and the subsequent inactivation of PI3K-Akt and dephosphorylation of FoxO3a and downregulation of SREBP-1 and ChREBP-1, which reverses renal lipid accumulation, apoptotic renal injury and oxidative stress.

PPARα activation leads to an increase in fatty acid catabolism and ATP production as well as a decrease in the levels of cytotoxic fatty acid peroxidation products and the promotion of cell viability and inhibition of renal epithelium cell death [24]. We have previously reported that a lack of PPARα activity in diabetic PPARα knockout mice and a deficiency of PPARα activity in *db/db* mice are associated with an increased renal accumulation of free fatty acids and triglycerides which contributes to lipotoxicity in the kidney [9], [10]. Furthermore, PPARα is indirectly involved in lipid metabolism by promoting AMPK phosphorylation [25]. *In vitro* studies demonstrated that fenofibrate activates AMPK in various cells, such as myocytes, retinal endothelial cells and human umbilical vein endothelial cells [26]–[28]. AMPK signaling also inhibits the inflammatory response, which is mediated by several transcription factors that are the downstream targets of AMPK, such as silent information regulator T1 (SIRT1), PGC-1α, p53 and FoxOs factors [29]–[33]. Recently we demonstrated that the AMPK activator resveratrol improves lipotoxicity in the kidney by enhancing the PPARα-ERR-1α-PPARα mediated removal of lipid accumulation and decreasing fatty acid biosynthesis, apoptotic renal cell death and oxidative stress related to FoxO3a dephosphorylation [34]. In the current study we have demonstrated that the activation of PPARα by fenofibrate directly activates AMPK and prevents renal lipid accumulation and renal cell injury in the diabetic mice and cultured mesangial cells treated with high-glucose media. In addition, in this study, diabetic condition (HbA1c level) improved with fenofibrate. Therefore, we cannot rule out the possibility that the improvement of diabetic condition might have affected other molecules, including SIRT1 and PPARγ, which play key roles in lipotoxicity in the kidney.

Interestingly, our study also showed that another critical mediator for the effects of PPARα on lipotoxicity is PGC-1α, which is a co-activator protein that interacts with several transcription factors, including ERR-1α and FoxOs. Like FoxOs, PGC-1α promotes the transcription of antioxidant enzymes in response to oxidative stress [35], [36]. The interactions of PGC-1α with ERR-1α are of unique importance. ERR-1α, activated by PGC-1α, induces genes and acts as a downstream effecter of PGC-1α in the regulation of expression of genes with roles in fatty acid oxidation, the TCA cycle, oxidative stress responses, mitochondrial biogenesis and dynamics and oxidative phosphorylation [14], [15], [37], [38]. It acts both directly on genes encoding mitochondrial structural components and enzymes, as well as indirectly by enhancing the expression of other transcriptional factors, such as NRF1 and NRF2/GABP, which regulate these pathways [39], [40].

Overexpression of lipogenic enzymes in the kidney are reported to be one cause of lipid accumulation and subsequent tissue damage including renal injury. Thus the regulation of enzyme expression associated with lipid metabolism may be a suitable strategy against lipotoxicity-associated tissue dysfunction. A recent study also demonstrated that the increases in SREBP-1- and ChREBP-1-dependent fatty acid synthesis pathways, which are responsible for converting excess carbohydrate to fatty acid for long-term storage, are paralleled by markedly decreased PPAR-α, PPAR-δ, and their target enzyme, acyl-CoA oxidase, which mediate fatty acid oxidation. It has been known that SREBP-1c is strongly linked to diabetic nephropathy, in which hyperglycemia activates mesangial SREBP-1c, ROS and mesangial proliferation and glomerular fibrosis [34], [41], [42]. In the current study, fenofibrate decreased SREBP-1 and ChREBP-1 and increased phosphorylation of ACC, which correlated with decreased lipid contents in the kidney. We also demonstrated that high-glucose treatment of mesangial cells induced the expression of genes involved in fatty acid synthesis, such as those encoding SREBP-1 and ChREBP-1. Fenofibrate reversed all of these changes, suggesting that the renoprotective impact of fenofibrate may be mediated by shifting the gene expression profile of the cells to a state that inhibits lipogenesis.

To date it has been known that PGC-1α is inactivated by the phosphorylation of Akt and Akt can have a negative impact on the PGC-1α expression via inactivation of the FoxOs factors, FoxO1 and FoxO3a which act as transcriptional regulators of PGC-1α expression [43], [44]. In obese or diabetic states, the FoxO1- and FoxO3a-dependent gene expression promotes some of the deleterious characteristics associated with hyperglycemia, glucose intolerance and lipotoxicity [43], [44]. Currently, it is not known whether there are differences in the activation levels of separate FoxO factors, particularly in different tissues. The renal expression of phospho-Ser^256^ FoxO1 and phosphor-Ser^253^ FoxO3a by fenofibrate treatment in overfed spontaneously hypertensive rats showed that phospho-Ser^253^ FoxO3a was strongly expressed in the cytoplasm of podocytes in the glomerulus and the collecting tubules, while phospho-Ser^256^ FoxO1 was strongly expressed in the proximal tubules and very weakly expressed in the glomerulus in the immunohistochemical staining [45]. In this study, fenofibrate treatment markedly decreased the renal expression of phosphor-Ser^253^ FoxO3a, whereas it did not have any effect on the phospho-Ser^256^ FoxO1 expression in the diabetic mice. The inactivation of PI3K/Akt and the activation of FoxO3a were one of the main pathways to reduce lipoapoptosis and oxidative stress as represented by the decrease of urinary 8-epi PDF_2α_ levels, which resulted in increases of anti-apoptotic BCL-2 and the antioxidants and a decrease of the pro-apoptotic gene BAX in diabetic mice.

Interestingly, there is a strong overlap in the genes transcriptionally regulated by AMPK and those by PGC-1α, hence suggesting that PGC-1α might be an important mediator AMPK-induced gene expression. Supporting this hypothesis, AMPK activation leads to increased PGC-1α expression [46], and AMPK requires PGC-1α activity to modulate the expression of several key players in mitochondrial and glucose metabolism [47]. In the current study, when AMPK was knocked down in mesangial cells, both AMPK–PGC-1α were also reduced. In contrast, when PGC-1α was knocked down, levels of PGC-1α were reduced, although AMPK was not. These results suggest that AMPK regulates the downstream PGC-1α in the mesangial cells.

In conclusion, this study demonstrated that activation of PPARα by fenofibrate was associated with the activation of AMPK-PGC-1α and a subsequent increase in ERR-1α expression, which consequently ameliorated lipotoxicity related to a decrease in lipogenesis-related SREBP-1 and ChREBP-1 and an increase in pACC expression in diabetic kidneys. Fenofibrate also inhibited activation of PI3K, phosphorylation of Akt, and phosphorylation of FoxO3a, resulting in the prevention of apoptotic renal cell death and oxidative stress. These results suggest that fenofibrate may be a potential therapeutic modality to modulate intra-renal AMPK-PGC-1α and FoxO3s signaling to treat type 2 diabetic nephropathy.
