# Correction: Fenofibrate Improves Renal Lipotoxicity through Activation of AMPK-PGC-1α in *db/db* Mice

**DOI:** 10.1371/journal.pone.0105921

**Published:** 2014-08-11

**Authors:** The PLOS ONE Staff

There is an error in the fourth sentence of the “Experimental methods” section of the Materials and Methods. The corrected sentence should read: In total, 20 mg/kg/day of fenofibrate was administered to the treated *db/db* and *db/m* groups.

There are errors in the sixth sentence of the second paragraph of the Discussion. The corrected sentence should read: Recently, we demonstrated that the AMPK activator resveratrol improves lipotoxicity in the kidney by enhancing the PPARα-ERR-1α-SREBP-1-mediated removal of lipid accumulation and decreasing apoptotic renal cell death and oxidative stress related to FoxO3a dephosphorylation in diabetic nephropathy.
